# Comparative analysis of COPD associated with tobacco smoking, biomass smoke exposure or both

**DOI:** 10.1186/s12931-018-0718-y

**Published:** 2018-01-18

**Authors:** Jordi Olloquequi, Sergio Jaime, Viviana Parra, Elizabeth Cornejo-Córdova, Gonzalo Valdivia, Àlvar Agustí, Rafael Silva O.

**Affiliations:** 1 Instituto de Ciencias Biomédicas, Facultad de Ciencias de la Salud, Universidad Autónoma de Chile, 5 Poniente #1670, 3460000 Talca, Chile; 2 Unidad Respiratorio, Centro de Diagnóstico Terapéutico, Hospital Regional de Talca, 1 Norte #1990, 3460000 Talca, Chile; 3 Subdepartamento de Salud Laboral, Hospital Regional de Talca, 1 Norte #1990, 3460000 Talca, Chile; 4 Departamento de Salud Pública, Facultad de Medicina, Pontificia Universidad Católica de Chile, Av. Libertador Bernardo O’Higgins #340, 35420000 Santiago, Chile; 5 Respiratory Institute, Hospital Clínic, Institut d’Investigacions Biomédiques August Pi i Sunyer (IDIBAPS) Universitat de Barcelona, Rosselló #149-153, 08036 Barcelona, Catalonia Spain

**Keywords:** COPD, Biomass smoke, Immunoglobulin E, Oxygen saturation, Systemic inflammation, Indoor pollution

## Abstract

**Background:**

Exposure to noxious gases and particles contained in both tobacco smoking (TS) and biomass smoke (BS) are well recognized environmental risk factors for chronic obstructive pulmonary disease (COPD). COPD is characterized by an abnormal inflammatory response, both in the pulmonary and systemic compartments. The differential effects of TS, BS or their combined exposure have not been well characterized yet. This study sought to compare the lung function characteristics and systemic inflammatory response in COPD patients exposed to TS, BS or their combination.

**Methods:**

Sociodemographic, clinical and lung functional parameters were compared across 49 COPD patients with a history of smoking and no BS exposure (TS COPD), 31 never-smoker COPD patients with BS exposure (BS COPD), 46 COPD patients with a combined exposure (TS + BS COPD) and 52 healthy controls (HC) who have never been exposed neither to TS or BS. Blood cell counts, C-reactive protein (CRP), fibrinogen and immunoglobulin E (IgE) levels were quantified in all four groups.

**Results:**

TS + BS COPD patients exhibited significantly lower oxygen saturation than the rest of groups (*p* < 0.01). Spirometry and diffusing capacity were significantly higher in BS than in TS or TS + BS patients. CRP levels were significantly higher in TS COPD patients than in BS COPD group (*p* < 0.05), whereas fibrinogen was raised in COPD patients with a history of smoking (TS and TS + BS) when compared to control subjects (*p* < 0.01). Finally, COPD patients with BS exposure (BS and BS + TS groups) showed higher IgE levels than TS and HC (*p* < 0.05).

**Conclusions:**

There are significant physiological and inflammatory differences between COPD patients with TS, BS and TS + BS exposures. The latter had worse blood oxygenation, whereas the raised levels of IgE in BS exposed patients suggests a differential Th2 systemic inflammatory pattern triggered by this pollutant.

## Background

COPD is characterized by an abnormal response of the lungs to toxic particles and gases, resulting in a progressive and not fully reversible airway obstruction whose main pathologic hallmarks are bronchiolitis and lung parenchymal emphysematous destruction [1]. Despite tobacco smoke (TS) is the environmental risk factor most frequently and clearly associated to COPD, exposure to biomass smoke (BS) has also been proposed as one of the main risk factors for developing the disease, especially among non-smokers [2, 3]. In this respect, the existing evidence of clinical, functional, radiologic and histopathologic differences between TS and BS-exposed COPD patients has raised the hypothesis of two distinct phenotypes of COPD, according to the main triggering risk factor. Thus, in addition to the known gender difference on exposure chance, BS COPD patients exhibit a milder airflow obstruction –measured by FEV_1_ and FEV_1_/FVC– than TS COPD subjects, as well as a higher DL_CO_ values [4–7]. On the other hand, BS COPD patients show more anthracosis and pulmonary fibrosis and less emphysema than TS COPD patients [8–10].

The evidence of different pathologic features in COPD patients exposed to BS or TS underpin the existence of a third potential phenotype which remains largely uncharacterized: COPD patients with a significant exposure to both TS and BS (TS + BS COPD). Given the toxicity of both kinds of smoke, a higher severity of pulmonary damage could be expected in double-exposed subjects. Following this line of thought, Dogan et al. [11] compared lung histopathological alterations among rats exposed exclusively to TS, those exclusively exposed to BS and a group with combined exposure to TS and BS. They found an increased severity in perivascular inflammation, as well as higher parenchymal infiltration, number of alveolar macrophages and vascular wall thickness in lungs of rats exposed to both BS and TS than in those only exposed to BS [11]. In turn, with data from the PREPOCOL study [12], Torres-Duque et al. [13] compared some demographical and functional parameters among 114 COPD patients exposed to wood smoke, 81 COPD patients exposed to TS and 264 COPD patients exposed to both types of smoke, reporting significant decreases in FEV_1_ and FEV_1_/FVC values in patients with a combined exposure [13]. In both studies, the authors suggested that a combined exposure to TS and BS could have a noxious additive effect in COPD patients and could increase the frequency of COPD symptoms.

On another front, although COPD is primarily a disease of the lungs and airways, it also has several extra-pulmonary manifestations and comorbidities. Indeed, systemic inflammation is a hallmark of COPD and it may be involved in cardiovascular complications in COPD patients [14]. Accordingly, increased numbers of inflammatory cells have been reported not only in small airways and lung parenchyma of COPD patients [15–17], but also in peripheral blood [18, 19]. Moreover, several molecular inflammatory markers are also increased in blood of COPD patients. One of the most widely studied is C-reactive protein (CRP), an acute-phase reactant secreted by the liver during inflammatory processes, which is increased in both stable and exacerbated COPD patients [20]. This biomarker has also proved to predict mortality outcomes in subjects with COPD [21]. COPD patients show higher blood levels of other inflammatory markers, such as interleukine (IL)-6, IL-8, tumor necrosis factor (TNF)-α and fibrinogen when compared to control subjects [22, 23]. Regarding the last one, it has been shown that fibrinogen is associated with disease severity and can also predict risk of exacerbations and mortality in COPD patients, thus highlighting its importance in predicting clinical outcomes [24, 25]. Notwithstanding, the role of other classical biomarkers of inflammation in COPD remain elusive. This is case of immunoglobulin E (IgE), which is mostly associated with asthma-COPD overlap syndrome (ACOS) [26], although it has also been shown to be increased in non-atopic COPD patients [27]. In spite of the importance of systemic inflammatory biomarkers in the understanding of COPD, data on systemic inflammation profile in BS COPD is scarce and no study has assessed these biomarkers in TS + BS COPD patients so far.

The purpose of the present study is to elucidate whether there are differences in clinical, functional and systemic inflammatory markers among TS, BS and TS + BS COPD patients.

## Methods

### Study population

We studied 126 COPD patients and 52 healthy volunteers recruited at Hospital Regional de Talca, Talca, Chile. The Ethics Committees of Maulean Health Service and Universidad Autónoma de Chile approved the study and written informed consent was obtained from all the subjects.

COPD patients were recruited at the Respiratory Service of the Hospital, where they attended to undergo diagnostic tests after suspected COPD or for COPD monitoring visits. Control subjects were recruited in parallel at the same ward through a volunteer recruitment program. After clinical characterization and risk factors assessment, subjects were classified into four groups: 49 TS COPD patients (ex-smokers with no BS exposure), 31 BS COPD patients (never-smokers with BS exposure), 46 TS + BS COPD patients (subjects with a combined exposure to TS and BS) and 52 controls, consisting in subjects with no record of any specific illness and with neither smoking history nor biomass smoke exposure.

Diagnostic evaluation of subjects was conducted according to GOLD criteria [1] and medical history was considered standardizing clinical information. Standard procedures [28] and equipment (Masterlab; Jaeger, Würzburg, Germany) were used to assess pulmonary function in all patients, including measurements of forced expiratory volume in 1 s (FEV_1_), forced vital capacity (FVC) and carbon monoxide diffusing capacity of the lung (DL_CO_). Oxygen saturation was measured in all patients by pulse-oximetry (Ohmeda TuffSat, Soma Technology, Connecticut, USA). Body mass index (BMI) was calculated using the current weight and the tallest height of each study participant. Dyspnea was determined using the modified Medical Research Council scale (mMRC) and exercise capacity was determined with the distance walked in 6 min test (6MWT). Health-related quality of life and symptom burden in patients with COPD was quantified using the COPD assessment test (CAT). A Body-mass, airflow Obstruction, Dyspnea and Exercise (BODE) score was assigned to each COPD patient. Subjects were excluded from the study when they presented a history of asthma, rhinitis or any extra-pulmonary disease affecting lung function, and when they had a positive bronchodilator test, FEV_1_ increasing by ≥12% and 200 ml**.** In order to ensure that COPD patients were stable, those with a COPD exacerbation or hospitalization record during the previous two months were also excluded from the study.

### Risk factors

Risk factors for COPD were considered to be exposure to either BS or TS as determined by the clinical interview and a standardized Spanish version of the CanCOLD study questionnaire [29] with the addition of some questions referring to biomass fuels.

For BS exposure assessment, specifically, subjects were asked the following two questions: 1) Has an indoor open fire with coal, coke, wood, pellet, agricultural residue or animal dung been used in your home as a primary means of cooking or heating for more than 6 months in your life? 2) For how many years? Subjects who answered affirmatively to first question and 9 or more years to the second question were considered as BS exposed. Cumulative exposure to biomass smoke was calculated and expressed as hour-years as previously described [4]. The amount of cigarette smoking history was measured by pack-years.

### Systemic inflammatory markers

A Beckman Coulter LH 750 analyzer (Beckman Coulter Inc., California, USA) was used to assess the blood cell counts. Serum CRP and IgE levels were measured by immunoturbidimetry and electrochemiluminescence immunoassay, respectively, using a Cobas 6000 analyzer (Roche Diagnostics, Basel, Switzerland). Plasma fibrinogen was measured by the Clauss method using the STA-Fibrinogen reagent and the STA-R evolution coagulation analyzer (Roche Diagnostics, Basel, Switzerland).

### Statistical analysis

Data are expressed as mean ± standard deviation or median (range) according to whether they conform to normal distribution. Differences among groups were analyzed using the analysis of variance (ANOVA) for parametric data (demographic, clinical and biomarkers data) and the Kruskall-Wallis test for non-parametric data (blood cell counts). When differences in cell counts were significant, the Kruskal-Wallis test was followed by the Mann-Whitney U test for comparison between groups. Correlations were calculated by Spearman’s rank correlation test. Significance level was set at *p* < 0.05. All analyses were performed using R statistical pack (R Foundation for Statistical Computing, Vienna, Austria).

## Results

### Demographic and clinical findings

Table 1 presents the main clinical and functional characteristics of study subjects. The Chi-square test revealed significant differences in gender proportions between control subjects and smoking COPD groups (TS and TS + BS) as well as between TS COPD and BS COPD (*p* < 0.05). The four groups had similar age. TS and BS exposures were similar in TS COPD and TS + BS COPD and BS COPD and TS + BS COPD, respectively. HS completed a significantly higher number of schooling years as compared to COPD patients. Furthermore, TS COPD patients showed a significantly higher average of schooling years when compared to BS-exposed groups. HC also had a significantly higher BMI value than COPD patients. There were no significant differences in the number of exacerbations in the previous year among COPD groups.

**Table 1:** Demographic and clinical data

	Control Subjects*n* = 52	TS COPD*n* = 49	BS COPD*n* = 31	TS + BS COPD *n* = 46
Sex, Male (%)/Female (%)	15(29)/37(71)	35(71)/14(29)	12(39)/19(61)	26(63)/20(37)
Age, years	70.34 ± 5.95	69.41 ± 8.69	72.29 ± 9.49	69.93 ± 7.19
Smoking history, pack-years	–	41.57 ± 25.62	–	55.46 ± 47.12
Biomass exposure, hour-years	–	–	340.90 ± 206.09	345.15 ± 193.16
Scolarship, years	13.21 ± 2.06	7.56 ± 4.25^a^	5.20 ± 3.59^a, b^	6.09 ± 3.93^a, b^
BMI, kg/m^2^	30.09 ± 5.54	27.67 ± 5.08^a^	26.57 ± 3.06^a^	27.35 ± 5.69^a^
Exacerbations in the previous year	–	1.10 ± 1.37	0.58 ± 0.42	0.69 ± 1.29
FEV_1_, % predicted	110.19 ± 17.46	56.88 ± 19.37^a, c^	68.09 ± 32.30^a^	53.79 ± 18.67^a, c^
FEV_1_/FVC, % predicted	103.87 ± 8.13	67.75 ± 14.00^a,c^	74.16 ± 10.69^a^	67.56 ± 15.97^a, c^
DL_CO_, % predicted	81.52 ± 21.21	66.60 ± 19.82^a^	73.73 ± 17.16	61.22 ± 24.98^a, c^
Oxygen Saturation, %	96.91 ± 1.36	92.65 ± 4.55^a^	93.94 ± 4.04^a^	90.52 ± 4.90^a, b, c^
6 MW, meters	492.48 ± 78.51	355.96 ± 163.02^a^	375.29 ± 143.75^a^	344.09 ± 161.12^a^
mMRC	–	2.38 ± 1.47	2.37 ± 1.16	2.67 ± 1.03
CAT	–	15.49 ± 8.21	14.84 ± 6.69	13.18 ± 6.69
BODE	–	2.88 ± 5.71	2.71 ± 6.35	3.87 ± 7.72

Data presented as mean ± standard deviation, unless otherwise indicated. Definition of abbreviations: *BMI* body-mass index, *FEV*_1_ forced expiratory volume in 1 s, *FVC* forced vital capacity. *DL*_CO_ carbon monoxide diffusing capacity, *6 MW* 6 min walking test, *mMRC* modified Medical Research Council scale *CAT* COPD assessment test, *BODE* Body-mass, airflow Obstruction, Dyspnea and Exercise index

^a^Different from control subjects (*p* < 0.05, by ANOVA)

^b^Different from TS COPD (*p* < 0.05, by ANOVA)

^c^Different from BS COPD (*p* < 0.05, by ANOVA)

### Lung function

COPD patients had a significantly reduced FEV_1_ and FEV_1_/FVC than HC. These parameters were significantly higher in BS than TS or double-exposed patients (Table 1). Likewise, BS COPD patients also showed a higher DL_CO_ than other COPD groups. Interestingly, oxygen saturation was significantly reduced in TS + BS COPD patients when compared to the rest of groups (*p* < 0.01). Finally, no differences among COPD groups were found in 6 MW test, mMRC, CAT and BODE index.

### Systemic inflammatory markers

**Fig. 1:**
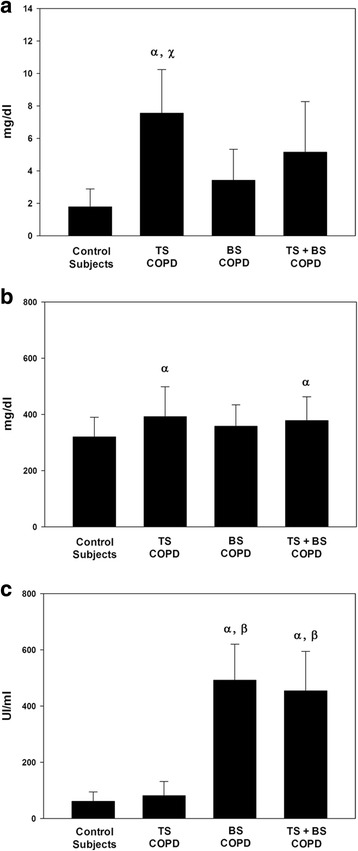
Quantification of systemic inflammatory markers. **a**) CRP levels, **b**) Fibrinogen levels, **c**) Ig E levels. ^α^Different from control subjects (*p* < 0.05). ^β^Different from TS COPD group (*p* < 0.05). ^χ^Different from BS COPD group (*p* < 0.05)

In order to determine whether the systemic inflammatory response was different among TS, BS and TS + BS COPD patients, blood levels of CRP, fibrinogen and immunoglobulin E were assessed (Fig. 1).

TS COPD patients showed a significantly higher level of CRP than BS COPD patients and control subjects (7.56 ± 2.68 vs 3.42 ± 1.90 and 1.79 ± 1.10 mg/l, respectively) (*p* < 0.05, Fig. 1a). Blood levels of fibrinogen were significantly increased in TS and TS + BS COPD patients when compared to control subjects (392.22 ± 106.38 and 378.85 ± 84.27 vs 319.81 ± 70.52 mg/dl) (*p* < 0.01, Fig. 1b). However, no significant differences in this biomarker were found between BS COPD and control subjects. Importantly, total IgE levels were significantly increased in BS and TB + BS COPD groups (492.87 ± 127.89 and 454.19 ± 140.69 UI/ml, respectively) as compared to TB COPD patients and control subjects (81.23 ± 50.34 and 60.57 ± 33.76 UI/ml, respectively) (*p* < 0.05, Fig. 1c).

On the other hand, there were no significant differences in erythrocytes, platelets and basophils among COPD groups (Table 2). However, COPD patients had higher numbers of neutrophils and lymphocytes than control subjects (*p* < 0.01). Furthermore, total leucocyte and monocyte counts were significantly higher in COPD patients with a history of smoking than in BS COPD and control subjects (*p* < 0.05 and *p* < 0.01, respectively, Table 2). Finally, the neutrophil-to-lymphocyte ratio (NLR) was significantly higher in COPD patients than control subjects (*p* < 0.01).

**Table 2:** Blood cell counts

	Control Subjects*n* = 52	TS COPD*n* = 49	BS COPD*n* = 31	TS + BS COPD *n* = 46
Eritrocytes, 1 × 10^6^ μl	4.53 (3.11–5.51)	4.64 (3.49–5.83)	4.54 (3.65–6.04)	4.79 (3.92–5.88)
Platelets, 1 × 10^3^ μl	251.00 (142.00–477.00)	251.00 (118.00–553.00)	243.00 (132.00–389.00)	236.00 (144.00–360.00)
Leucocytes, 1 × 10^3^ μl	7.40 (3.50–10.80)	8.80 (4.10–12.60)^a, c^	7.90 (4.10–11.70)	8.20 (5.30–11.80)^a, c^
Neutrophils, 1 × 10^3^ μl	4.20 (2.00–7.00)	5.60 (2.50–8.10)^a^	5.20 (2.20–7.80)^a^	4.90 (2.80–7.50)^a^
Eosinophils, 1 × 10^2^ μl	0.20 (0.00–0.30)	0.20 (0.00–0.30)	0.10 (0.00–0.60)	0.20 (0.00–0.70)
Basophils, 1 × 10 μl	0.50 (0.00–2.20)	0.40 (0.00–1.20)	0.50 (0.00–1.00)	0.50 (0.00–1.00)
Monocytes, 1 × 10^3^ μl	0.50 (0.20–0.70)	0.60 (0.40–6.00)^a, c^	0.50 (0.30–1.10)	0.60 (0.40–1.60)^a, c^
Lymphocytes, 1 × 10^3^ μl	2.38 (1.20–3.90)	2.06 (0.80–3.80)^a^	1.76 (1.00–2.80)^a^	2.11 (1.00–3.60)^a^
NLR	1.76 (0.87–3.00)	2.46 (1.36–5.14)^a^	2.94 (1.09–6.10)^a^	2.48 (0.88–4.18)^a^

Data presented as median (range). NLR: neutrophil-to-lymphocyte ratio

^a^Different from control subjects (*p* < 0.05, by Kruskall-Wallis test)

^b^Different from TS COPD (*p* < 0.05, by Kruskall-Wallis test)

^d^Different from BS COPD (*p* < 0.05, by Kruskall-Wallis test)

### Correlations of interest

Some significant correlations between clinical and haematological parameters were found when all COPD patients were considered as one group. An inverse correlation was found between this CRP and FEV_1_ (% of predicted) (*R* = −0.19, *p* < 0.05). FEV_1_ was also inversely correlated with total leucocyte counts (R = −0.19, *p* < 0.05), as well as with neutrophils (*R* = −0.24, *p* < 0.05) and monocytes (*R* = −0.27, *p* < 0.01) counts. Moreover, exercise capacity was inversely correlated with fibrinogen and IgE (*R* = −0.59, p < 0.01 and *R* = −0.63, p < 0.01, respectively) as well as with eosinophils (*R* = −0.47, *p* < 0.05). Regarding BODE index, it was significantly correlated with fibrinogen (*R* = 0.59, *p* < 0.01) and IgE (*R* = 0.53, *p* < 0.05).

With respect to exposure to risk factors, significant correlations were found between pack-years and eosinophils (*R* = 0.30, *p* < 0.05) and neutrophils (*R* = 0.27, *p* < 0.05) counts, as well as with NLR ratio (*R* = 0.26, *p* < 0.05). Moreover, the cumulative exposure to biomass smoke was significantly correlated with total blood leucocytes (*R* = 0.69, *p* < 0.01), neutrophils (*R* = 0.78, *p* < 0.01) and lymphocytes (*R* = 0.55, *p* < 0.05) counts, in addition to NLR ratio (*R* = 0.52, *p* < 0.05). Finally, no significant correlation was found between tobacco pack-years or biomass cumulative exposure and lung function parameters.

## Discussion

The results of this study show that there are significant clinical, functional and systemic differences between COPD patients exposed to TS, BS or both. In general, lung function was more severely impaired in COPD patients exposed to TS, who also showed higher levels of fibrinogen, circulating leucocytes and monocytes, whereas COPD patients exposed to BS had significantly higher levels of blood IgE. These results suggest different inflammatory responses to different inhaled pollutants in COPD.

### Previous studies

To our knowledge, this is the first article comparing clinical, functional and blood parameters among TS COPD patients, BS COPD patients, COPD patients exposed to both risk factors and healthy subjects never exposed to either of them.

A previous study presented at the American Thoracic Society 2013 International Conference by Torres-Duque et al. [13], however, compared the effects of the combined exposure to TS and wood smoke in lung function of COPD patients. They reported significant decreases in FEV_1_ and FEV_1_/FVC values in patients with a combined exposure. Conversely, we found that FEV_1_ and FEV_1_/FVC values were similar between TS and TS + BS COPD group and significantly lower than in BS group. Since no data about amount of exposure to TS and BS was available in the study of Torres-Duque et al. [13], the differences in the double exposed group may be due to a higher exposure to TS and/or BS in these patients than in those with a single exposure. Moreover, the contradictory results between the study of Torres-Duque et al. and the present one could also be explained by the fact that they only considered wood smoke exposure, whereas we considered different sources of BS.

### Interpretation of findings

Despite the Global Burden of Disease study reported 1.1 million COPD-related deaths for tobacco smoking and 850.000 to indoor pollution in 2010 [30], information about the characteristics of COPD patients with a combined exposure to both risk factors is virtually non-existent. The following novel observations emerge from our study:

First, although it is well established that lower socioeconomic status and education level is associated with COPD [31–33], we observed that the number of years of scholarship was significantly reduced in BS COPD and TS + BS COPD patients when compared to TS COPD patients. This is likely related to the fact that the use of biomass as a source of energy is more widespread in rural areas, where also the access to education is more difficult, especially some decades ago.

Second, arterial oxygen saturation was significantly lower in double exposed COPD patients, suggesting an increased ventilation/perfusion mismatch in these patients [34]. Bearing in mind that that both smoking [35] and exposure to some components of BS [36] have been related to a decrease in oxygen saturation, this result likely reflects an additive effect of both exposures, and it has important clinical implications, since an oxygen saturation cut-off value of ≤92% is considered when screening for respiratory failure in patients with COPD [37]. In spite of this, no other functional parameter was exclusively impaired in the TS + BS COPD group, albeit airflow limitation was more severe in TS COPD than in BS COPD patients, in keeping with some previous studies [4, 5, 7].

Third, CRP levels were significantly higher in TS than in BS COPD and HS. This contrasts with two previous studies reporting similar CRP levels in TS and wood-smoke exposed COPD patients [38, 39]. This discrepancy may be explained by the different techniques used in CRP quantification (ELISA and immunoturbidimetry) and the characteristics of the study populations. It’s worth to mention, however, that both in our study and in one of these previous ones [38] CRP levels were inversely related with FEV_1_ values, as it has been reported in other TS COPD studies [40, 41]. Likewise, circulating levels of fibrinogen were also significantly higher in TS and TS + BS COPD patients than in HC, in keeping with some previous studies also reporting higher levels of fibrinogen in smokers [42, 43].

Fourth, circulating IgE levels were significantly higher in COPD groups with a BS exposure (BS and TS + BS) than in TS COPD and HC. Although it has been shown that particulate matter (PM) and polycyclic aromatic hydrocarbons (PAHs) contained in BS increase blood IgE levels [44–46], this is the first study reporting an increase of this inflammatory marker specifically in COPD patients exposed to BS. Since an increase of IgE production represents a typical feature of a Th2 response, this result suggests that BS could trigger a specific inflammatory Th2 response in COPD patients. In fact, Solleiro-Villavicencio et al. reported a higher frequency of Th2 cells and a higher concentration of IL-2 in blood of BS vs. TS COPD patients and HC [47]. Although quantification of lymphocyte subsets was not available in our study, it is noteworthy that cumulative BS was significantly correlated with total blood lymphocyte counts. On the other hand, the increased IgE levels observed in BS COPD patients could also underlie the higher prevalence of ACOS reported in COPD subjects exposed to BS [7]. Moreover, the transversal nature of the present study does not allow ruling out the possibility of other exposures capable of raising Ig E levels, particularly in rural areas.

Of note, a previous study comparing IgE and fibrinogen between COPD patients exposed to TS and BS [39] reported similar blood levels of both biomarkers in both groups. We believe that this discrepancy can be explained by sample size, ethnic and sex differences [48] (75% of males in the cited study vs. 58% of males in the present one). Moreover, since the composition of BS depends on the type of fuel and the combustion conditions, BS exposure from different sources may lead to different clinical features.

Finally, our results showed significantly increased numbers of neutrophils, lymphocytes and NLR in all COPD groups vs. HC. This highlights a general a systemic inflammatory process mounted in response to the inhalation of noxious gases and particles in COPD, regardless of their specific type.

### Potential limitations

The present study has several potential limitations. Firstly, the study population was relatively small. However, we believe that the rigorous characterization of subjects allowed establishing reliable comparisons among groups. Secondly, imaging and histopathological analyses which would have allowed the assessment of small airways thickening and emphysema were not available. Thirdly, patterns of exposure to BS and TS may have biased our results, since COPD patients with a smoking history were actually ex-smokers, whereas several BS exposed subjects reported a current use of biomass fuels. Fourthly, although subjects with a history of asthma and/or reversibility in post-bronchodilator FEV_1_ were excluded from our study, we cannot rule out the possibility of ACOS among our patients, since no unified definition or diagnostic criteria for ACOS exists. Finally, given that subjects exposed to BS exhibited a lower socioeconomic status and an ancestry characterization was not available, both environmental and genetic variables may have been a potential source of bias.

## Conclusions

There is a synergistic damaging effect of the combined exposure to TB and BS. The systemic inflammatory response in these two types of COPD is, however, distinct. Given that BS and TS have many components in common, a further characterization at a molecular level would be necessary in order to elucidate which ones could be responsible of different inflammatory features, and in which concentrations.

## Abbreviations

6MWT: Six minutes walking test
ACOS: Asthma-COPD overlapping syndrome
BMI: Body mass index
BODE: Body-mass, airflow Obstruction, Dyspnea and Exercise score
BS: Biomass smoke
CAT: COPD assessment test
COPD: Chronic obstructive pulmonary disease
CRP: C-reactive protein
DLCO: Carbon monoxide diffusing capacity
FEV1: Forced expiratory volume in 1 s
FVC: Forced vital capacity
HC: Healthy controls
IgE: Immunoglobulin E
IL: Interleukine
mMRC: Modified Medical Research Council scale
NLR: Neutrophil-to-lymphocyte ratio
TNF: Tumor necrosis factor
TS: Tobacco smoke

## References

[1] Global Initiative for Chronic Obstructive Lung Disease (GOLD). Global Strategy for the Diagnosis, Management and prevention of COPD. 2017. http://www.goldcopd.org/. Accessed 11 Sep 2017.

[2] Silva R, Oyarzun M, Olloquequi J (2015). Pathogenic mechanisms in chronic obstructive pulmonary disease due to biomass smoke exposure. Arch Bronconeumol.

[3] Olloquequi J, Silva OR (2016). Biomass smoke as a risk factor for chronic obstructive pulmonary disease: effects on innate immunity. Innate Immun.

[4] Ramirez-Venegas A, Sansores RH, Perez-Padilla R, Regalado J, Velazquez A, Sanchez C, Mayar ME (2006). Survival of patients with chronic obstructive pulmonary disease due to biomass smoke and tobacco. Am J Respir Crit Care Med.

[5] Ramirez-Venegas A, Sansores RH, Quintana-Carrillo RH, Velazquez-Uncal M, Hernandez-Zenteno RJ, Sanchez-Romero C, Velazquez-Montero A, Flores-Trujillo F (2014). FEV1 decline in patients with chronic obstructive pulmonary disease associated with biomass exposure. Am J Respir Crit Care Med.

[6] Gonzalez-Garcia M, Maldonado Gomez D, Torres-Duque CA, Barrero M, Jaramillo Villegas C, Perez JM, Varon H (2013). Tomographic and functional findings in severe COPD: comparison between the wood smoke-related and smoking-related disease. J Bras Pneumol.

[7] Golpe R, Sanjuan Lopez P, Cano Jimenez E, Castro Anon O, Perez de Llano LA (2014). Distribution of clinical phenotypes in patients with chronic obstructive pulmonary disease caused by biomass and tobacco smoke. Arch Bronconeumol.

[8] Camp PG, Ramirez-Venegas A, Sansores RH, Alva LF, McDougall JE, Sin DD, Pare PD, Muller NL, Silva CI, Rojas CE, Coxson HO (2014). COPD phenotypes in biomass smoke- versus tobacco smoke-exposed Mexican women. Eur Respir J.

[9] Rivera RM, Cosio MG, Ghezzo H, Salazar M, Perez-Padilla R (2008). Comparison of lung morphology in COPD secondary to cigarette and biomass smoke. Int J Tuberc Lung Dis.

[10] Moreira MAC, Barbosa MA, McdCAM DQ, KISS T, Pptes T, de Santana PJ, Montadon ME, Jardim JR (2013). Pulmonary changes on HRCT scans in nonsmoking females with COPD due to wood smoke exposure. Jornal Brasileiro de Pneumologia : Publicaça̋o Oficial da Sociedade Brasileira de Pneumologia e Tisilogia.

[11] Dogan OT, Elagoz S, Ozsahin SL, Epozturk K, Tuncer E, Akkurt I (2011). Pulmonary toxicity of chronic exposure to tobacco and biomass smoke in rats. Clinics.

[12] Caballero A, Torres-Duque CA, Jaramillo C, Bolivar F, Sanabria F, Osorio P, Orduz C, Guevara DP, Maldonado D (2008). Prevalence of COPD in five Colombian cities situated at low, medium, and high altitude (PREPOCOL study). Chest.

[13] Torres-Duque CA CA, González-García M, Jaramillo C, Maldonado D: Chronic Obstructive Pulmonary Disease In People Exposed To Wood Smoke. PREPOCOL: A Population Based. In *American Thoracic Society* 2013 *International Conference* (ATS ed., vol. 187. pp. A364. Philadelphia: Am J Respir Crit Care Med; 2013:A364.

[14] Sin DD, Man SF (2003). Why are patients with chronic obstructive pulmonary disease at increased risk of cardiovascular diseases? The potential role of systemic inflammation in chronic obstructive pulmonary disease. Circulation.

[15] Olloquequi J, Ferrer J, Montes JF, Rodriguez E, Montero MA, Garcia-Valero J (2010). Differential lymphocyte infiltration in small airways and lung parenchyma in COPD patients. Respir Med.

[16] Olloquequi J, Montes JF, Prats A, Rodriguez E, Montero MA, Garcia-Valero J, Ferrer J (2011). Significant increase of CD57+ cells in pulmonary lymphoid follicles of COPD patients. Eur Respir J.

[17] Olloquequi J, Garcia-Valero J, Rodriguez E, Montero MA, Ferrer J, Montes JF (2012). Lung CD57+ cell density is increased in very severe COPD. Histol Histopathol.

[18] Lewis SA, Pavord ID, Stringer JR, Knox AJ, Weiss ST, Britton JR (2001). The relation between peripheral blood leukocyte counts and respiratory symptoms, atopy, lung function, and airway responsiveness in adults. Chest.

[19] Vaguliene N, Zemaitis M, Lavinskiene S, Miliauskas S, Sakalauskas R (2013). Local and systemic neutrophilic inflammation in patients with lung cancer and chronic obstructive pulmonary disease. BMC Immunol.

[20] Zhang Y, Bunjhoo H, Xiong W, Xu Y, Yang D (2012). Association between C-reactive protein concentration and chronic obstructive pulmonary disease: a systematic review and meta-analysis. J Int Med Res.

[21] Kelly E, Owen CA, Pinto-Plata V, Celli BR (2013). The role of systemic inflammatory biomarkers to predict mortality in chronic obstructive pulmonary disease. Expert Rev Respir Med.

[22] Gan WQ, Man SF, Senthilselvan A, Sin DD (2004). Association between chronic obstructive pulmonary disease and systemic inflammation: a systematic review and a meta-analysis. Thorax.

[23] Su B, Liu T, Fan H, Chen F, Ding H, Wu Z, Wang H, Hou S (2016). Inflammatory markers and the risk of chronic obstructive pulmonary disease: a systematic review and meta-analysis. PLoS One.

[24] Duvoix A, Dickens J, Haq I, Mannino D, Miller B, Tal-Singer R, Lomas DA (2013). Blood fibrinogen as a biomarker of chronic obstructive pulmonary disease. Thorax.

[25] Mannino DM, Tal-Singer R, Lomas DA, Vestbo J, Graham Barr R, Tetzlaff K, Lowings M, Rennard SI, Snyder J, Goldman M (2015). Plasma fibrinogen as a biomarker for mortality and hospitalized exacerbations in people with COPD. Chronic Obstr Pulm Dis.

[26] Kobayashi S, Hanagama M, Yamanda S, Ishida M, Yanai M (2016). Inflammatory biomarkers in asthma-COPD overlap syndrome. Int J Chron Obstruct Pulmon Dis.

[27] Samaha HMS, Elsaid AR, NasrEldin E (2015). Total serum IgE level in COPD patients Egyptian. Journal of Chest Diseases and Tuberculosis.

[28] Crapo RHJ, Irvin C. Standardization of Spirometry, 1994 Update. American Thoracic Societye. Am J Respir Crit Care Med. 1995(152):1107–36. 10.1164/ajrccm.152.3.7663792 7663792

[29] Tan WC, Sin DD, Bourbeau J, Hernandez P, Chapman KR, Cowie R, JM FG. Characteristics of COPD in never-smokers and ever-smokers in the general population: results from the CanCOLD study. 2015;70:822–9. 10.1136/thoraxjnl-2015-206938 26048404

[30] Institute for Health Metrics and Evaluation. Global burden of disease study 2010 (GBD 2010) data downloads. 2013. http://ghdx.healthdata.org/record/global-burden-disease-study-2010-gbd-2010-mortality-results-1970-2010. Accessed 01 Oct 2017.

[31] Kanervisto M, Vasankari T, Laitinen T, Heliövaara M, Jousilahti P, Saarelainen S (2011). Low socioeconomic status is associated with chronic obstructive airway diseases. Respir Med.

[32] Bakke PS, Hanoa R, Gulsvik A (1995). Educational level and obstructive lung disease given smoking habits and occupational airborne exposure: a Norwegian community study. Am J Epidemiol.

[33] Yin P, Zhang M, Li Y, Jiang Y, Zhao W: Prevalence of COPD and its association with socioeconomic status in China: findings from China chronic disease risk factor surveillance 2007**.** BMC Public Health 2011, 11**:**586–586. 10.1186/1471-2458-11-586 PMC3152537 21781320

[34] Kent BD, Mitchell PD, McNicholas WT (2011). Hypoxemia in patients with COPD: cause, effects, and disease progression. Int J Chron Obstruct Pulmon Dis.

[35] Vold ML, Aasebo U, Hjalmarsen A, Melbye H (2012). Predictors of oxygen saturation </=95% in a cross-sectional population based survey. Respir Med.

[36] Lee KY, Chiang LL, Ho SC, Liu WT, Chen TT, Feng PH, Su CL, Chuang KJ, Chang CC, Chuang HC (2016). Associations of autophagy with lung diffusion capacity and oxygen saturation in severe COPD: effects of particulate air pollution. Int J Chron Obstruct Pulmon Dis.

[37] Roberts CM, Bugler JR, Melchor R, Hetzel MR, Spiro SG (1993). Value of pulse oximetry in screening for long-term oxygen therapy requirement. Eur Respir J.

[38] Montaño M, Sansores RH, Becerril C, Cisneros J, González-Avila G, Sommer B, Ochoa L, Herrera I, Ramírez-Venegas A, Ramos C: FEV(1) inversely correlates with metalloproteinases 1, 7, 9 and CRP in COPD by biomass smoke exposure**.** Respir Res 2014, 15**:**74–74. 10.1186/1465-9921-15-74 PMC4086695 24980707

[39] Golpe R, Martín-Robles I, Sanjuán-López P, Pérez-de-Llano L, González-Juanatey C, López-Campos JL, Arellano-Orden E (2017). Differences in systemic inflammation between cigarette and biomass smoke-induced COPD. Int J Chron Obstruct Pulmon Dis.

[40] de Torres JP, Cordoba-Lanus E, Lopez-Aguilar C, Muros de Fuentes M, Montejo de Garcini A, Aguirre-Jaime A, Celli BR, Casanova C (2006). C-reactive protein levels and clinically important predictive outcomes in stable COPD patients. Eur Respir J.

[41] Agarwal R, Zaheer MS, Ahmad Z, Akhtar J: The relationship between C-reactive protein and prognostic factors in chronic obstructive pulmonary disease**.** Multidisciplinary Respir Med 2013, 8**:**63–63. 10.1186/2049-6958-8-63 PMC3844686 24073993

[42] Lowe GD (2001). Why do smokers have higher plasma fibrinogen levels than non-smokers?. Clin Sci (Lond).

[43] Sinha S, Luben RN, Welch A, Bingham S, Wareham NJ, Day NE, Khaw KT (2005). Fibrinogen and cigarette smoking in men and women in the European prospective investigation into cancer in Norfolk (EPIC-Norfolk) population. Eur J Cardiovasc Prev Rehabil.

[44] Takafuji S, Suzuki S, Koizumi K, Tadokoro K, Ohashi H, Muranaka M, Miyamoto T (1989). Enhancing effect of suspended particulate matter on the IgE antibody production in mice. Int Arch Allergy Appl Immunol.

[45] Herr CE, Ghosh R, Dostal M, Skokanova V, Ashwood P, Lipsett M, Joad JP, Pinkerton KE, Yap PS, Frost JD (2011). Exposure to air pollution in critical prenatal time windows and IgE levels in newborns. Pediatr Allergy Immunol.

[46] Samuelsen M, Nygaard UC, Lovik M (2008). Allergy adjuvant effect of particles from wood smoke and road traffic. Toxicology.

[47] Solleiro-Villavicencio H, Quintana-Carrillo R, Falfan-Valencia R, Vargas-Rojas MI (2015). Chronic obstructive pulmonary disease induced by exposure to biomass smoke is associated with a Th2 cytokine production profile. Clin Immunol.

[48] Faner R, Gonzalez N, Cruz T, Kalko SG, Agusti A (2014). Systemic inflammatory response to smoking in chronic obstructive pulmonary disease: evidence of a gender effect. PLoS One.

