# Correction to: Comparative analysis of COPD associated with tobacco smoking, biomass smoke exposure or both

**DOI:** 10.1186/s12931-018-0765-4

**Published:** 2018-04-30

**Authors:** Jordi Olloquequi, Sergio Jaime, Viviana Parra, Elizabeth Cornejo-Córdova, Gonzalo Valdivia, Àlvar Agustí, Rafael O. Silva

**Affiliations:** 1 Instituto de Ciencias Biomédicas, Facultad de Ciencias de la Salud, Universidad Autónoma de Chile, 5 Poniente #1670, 3460000 Talca, Chile; 2 Unidad Respiratorio, Centro de Diagnóstico Terapéutico, Hospital Regional de Talca, 1 Norte #1990, 3460000 Talca, Chile; 3 Subdepartamento de Salud Laboral, Hospital Regional de Talca, 1 Norte #1990, 3460000 Talca, Chile; 4 Departamento de Salud Pública, Facultad de Medicina, Pontificia Universidad Católica de Chile, Av. Libertador Bernardo O’Higgins #340, 35420000 Santiago, Chile; 5 Respiratory Institute, Hospital Clínic, Institut d’Investigacions Biomédiques August Pi i Sunyer (IDIBAPS) Universitat de Barcelona, Rosselló #149-153, 08036 Barcelona, Catalonia Spain

## Correction

In the original publication [1] there is an error in the FEV1/FVC values found in Table 1. The correct version can be found in this Erratum.

**Table 1:** Demographic and clinical data

	Control Subjects *n* = 52	TS COPD *n* = 49	BS COPD *n* = 31	TS + BS COPD *n* = 46
Sex, Male (%)/Female (%)	15(29) / 37(71)	35(71) / 14(29)	12(39) / 19(61)	26(63) / 20(37)
Age, years	70.34 ± 5.95	69.41 ± 8.69	72.29 ± 9.49	69.93 ± 7.19
Smoking history, pack-years	–	41.57 ± 25.62	–	55.46 ± 47.12
Biomass exposure, hour-years	–	–	340.90 ± 206.09	345.15 ± 193.16
Scolarship, years	13.21 ± 2.06	7.56 ± 4.25 ^a^	5.20 ± 3.59 ^a, b^	6.09 ± 3.93 ^a, b^
BMI, kg/m^2^	30.09 ± 5.54	27.67 ± 5.08 ^a^	26.57 ± 3.06 ^a^	27.35 ± 5.69 ^a^
Exacerbations in the previous year	–	1.10 ± 1.37	0.58 ± 0.42	0.69 ± 1.29
FEV_1_, % predicted	110.19 ± 17.46	56.88 ± 19.37 ^a, c^	68.09 ± 32.30 ^a^	53.79 ± 18.67 ^a, c^
FEV_1_/FVC, %	84.46 ± 4.24	54.25 ± 11.17 ^a^	58.39 ± 8.62 ^a^	53.53 ± 12.35 ^a, c^
DL_CO_, % predicted	81.52 ± 21.21	66.60 ± 19.82 ^a^	73.73 ± 17.16	61.22 ± 24.98 ^a, c^
Oxygen Saturation, %	96.91 ± 1.36	92.65 ± 4.55 ^a^	93.94 ± 4.04 ^a^	90.52 ± 4.90 ^a, b, c^
6 MW, meters	492.48 ± 78.51	355.96 ± 163.02 ^a^	375.29 ± 143.75 ^a^	344.09 ± 161.12 ^a^
mMRC	–	2.38 ± 1.47	2.37 ± 1.16	2.67 ± 1.03
CAT	–	15.49 ± 8.21	14.84 ± 6.69	13.18 ± 6.69
BODE	–	2.88 ± 5.71	2.71 ± 6.35	3.87 ± 7.72

Data presented as mean ± standard deviation, unless otherwise indicated. Definition of abbreviations: *BMI* body-mass index, *FEV*_*1*_ forced expiratory volume in 1 s, *FVC* forced vital capacity, *DL*_*CO*_ carbon monoxide diffusing capacity, *6 MW* 6 min walking test, *mMRC* modified Medical Research Council scale, *CAT* COPD assessment test, *BODE* Body-mass, airflow Obstruction, Dyspnea and Exercise index

^a^Different from control subjects (*p* < 0.05, by ANOVA)

^b^Different from TS COPD (*p* < 0.05, by ANOVA)

^c^Different from BS COPD (*p* < 0.05, by ANOVA)
